# A fixed-point algorithm for estimating amplification efficiency from a polymerase chain reaction dilution series

**DOI:** 10.1186/s12859-014-0372-4

**Published:** 2014-12-10

**Authors:** Michael E Jones, George C Mayne, Tingting Wang, David I Watson, Damian J Hussey

**Affiliations:** Department of Anaesthesia and Pain Management, and Department of Anatomy, Flinders University, Bedford Park SA, 5042 Australia; Department of Surgery, Flinders University, Bedford Park SA, 5042 Australia

**Keywords:** qPCR, Fixed-point, Amplification efficiency

## Abstract

**Background:**

The polymerase chain reaction amplifies and quantifies small amounts of DNA. It is a cyclic process, during each cycle of which each strand of template DNA is copied with probability approaching one: the amount of DNA approximately doubles and this amount can be estimated fluorimetrically each cycle, producing a set of fluorescence values hereafter referred to as the amplification curve. Commonly the biological question of relevance is one of the ratio of DNA concentrations in two samples: a ratio that is deduced by comparing the two amplification curves, usually by way of a plot of fluorescence against cycle number. Central to this analysis is measuring the extent to which one amplification curve is shifted relative to the other, a measurement often accomplished by defining a threshold or quantification cycle, *C*_*q*_, for each curve: the fractional cycle number at which fluorescence reaches some threshold or at which some other criterion (maximum slope, maximum rate of change of slope) is satisfied.

We propose an alternative where position is measured relative to a reference curve; position equates to the cycle shift which maximizes the correlation between the reference and the observed fluorescence sequence. A key parameter of the reference curve is obtained by fixed-point convergence.

**Results:**

We consider the analysis of dilution series constructed for the estimation of qPCR amplification efficiency. The estimate of amplification efficiency is based on the slope of the regression line when the *C*_*q*_ is plotted against the logarithm of dilution. We compare the approach to three commonly used methods for determining *C*_*q*_; each is applied to publicly accessible calibration data sets, and to ten from our own laboratory. As in the established literature we judge their relative merits both from the standard deviation of the slope of the calibration curve, and from the variance in *C*_*q*_ for replicate fluorescence curves.

**Conclusions:**

The approach does not require modification of experimental protocols, and can be applied retrospectively to existing data. We recommend that it be added to the methodological toolkit with which laboratories interpret their real-time PCR data.

**Electronic supplementary material:**

The online version of this article (doi:10.1186/s12859-014-0372-4) contains supplementary material, which is available to authorized users.

## Background

Since its introduction by Mullis *et al.* [1], the polymerase chain reaction (PCR) has been widely used to amplify and quantify small amounts of DNA. Briefly it constitutes a few dozen cycles in each of which there are three stages: denaturation, annealing and extension.

At each cycle each DNA strand either doubles (with probability *p*) or fails to double. Defining amplification efficiency, *E*, as *E*=1+*p* the expected number *N*_*c*_ of strands after *C* cycles, given constant *p*, is

$$ N_{c} = N_{0}(1 + p)^{C} = N_{0} E^{C}, $$

where *N*_0_ is the initial number. We use this definition of *E* to be consistent with Ruitjer *et al.* [2] and in that we deviate from MIQE guidelines [3].

The amount of DNA can be estimated fluorimetrically using, for instance, Sybr Green 1 dye which binds to the minor groove in double-stranded DNA [4]. Commonly the relevant question biologically is one of relative quantification: the ratio of initial DNA in two samples. If samples *A* and *B*, initially containing *N*_*A*_ and *N*_*B*_ strands, exhibit the same fluorescence after *C*_*a*_ and *C*_*b*_ cycles respectively, then assuming constant *p*,

$$ N_{A}(1 + p)^{C_{a}} = N_{B}(1 + p)^{C_{b}}, $$

from which we have

(1) $$ N_{A}/N_{B} = (1+p)^{(C_{b} - C_{a})} = E^{\Delta C},  $$

where *ΔC*=*C*_*b*_−*C*_*a*_. Accordingly, the cycle difference, *ΔC*, and *E* (or equivalently, *p*), are the key to estimation of the ratio *N*_*A*_/*N*_*B*_.

In common use there are two fundamentally different approaches to the estimation of *E*. One involves the generation of curves from a series of dilutions: an eight-fold dilution, for instance, would delay the fluorescence by three cycles if *E*=2 because 2^3^=8. If *E*=1.8, however, it would delay the fluorescence curve by 3.53 cycles, because 1.8^3.53^=8. Alternatively (Gentle *et al.,* 2001. [5], Zhao and Fernald, 2005 [6]), *E* can be estimated from the real-time fluorescence data. At each cycle early in the sequence, fluorescence above background will increase by a factor *E*, which can therefore be estimated from the data in this ‘log-linear’ phase. The early phase of exponential increase is short-lived. As resources become limiting, the fluorescence curve flattens out, and Liu and Saint [7] have used the sigmoid or logistic function,

(2) $$ F(x) = F_{b} + \frac{F_{max}}{1 + e^{\beta(x_{0} - x)}},  $$

where *F*_*b*_ is background, and *F*_*max*_ is maximum contribution of the reaction to fluorescence, (the asymptote, rather than maximum observed experimentally), to describe the data. Rutledge and Stewart [8] introduced an analysis which takes into account the linear decrease in amplification under this model, simplifying the estimation of the initial amplification efficiency from the curve itself. MIQE guidelines [3] recommend the former approach: *‘PCR amplification efficiency must be established by means of calibration curves...’* but we acknowledge ongoing debate on this issue.

Strictly speaking the data from a tube are discontinuous; fluorescence is measured at the end of each cycle, and there is no such thing as a fluorescence after a fractional number of cycles as implied by the continuous functions above. We use the term reference curve to imply an abstraction; a smooth continuous curve of fluorescence as a function of *x*, which we observe at cycles *C* which are integer values of *x*. The observed fluorescence is the fluorescence at these integer values, but with the addition of error or noise.

A key to analyzing PCR, therefore, is, given two fluorescence curves, to measure *ΔC*, the extent to which one curve is shifted laterally relative to the other. There are two very different circumstances under which one may need to do this. If *E* is to be estimated from the cycle-to-cycle increase in fluorescence of a single assay tube, then quantifying some aspect of the fluorescence is important. Conversely, if one is using dilution to estimate amplification then the shape of the curve is of less import so long as the data and the reference curve have in common that they are S-shaped (sigmoid): interest lies only in the extent to which dilution has shifted the curve, of whatever shape, to the right. Whatever the method of estimating *E*, that estimate is commonly used subsequently to derive, in concert with a measured cycle difference between two tubes, *ΔC*, the initial concentration ratio implied by Eq. 1.

It is the estimation of cycle shift in these scenarios which we address; to what extent is one fluorescence curve shifted relative to another? There is, of course, a significant literature detailing several algorithms to do just that, and we should justify any attempt to add another. Ruitjer *et al.* [2] have examined the performance of nine estimators of *E* and have proposed several measures of their relative merits. In using the publicly available data sets comprising dilution series for establishing amplification efficiency, two measures are of central importance. One is the within-replicate variance; most data sets have three or more replicates at each dilution, and for a good estimator we expect values of *C*_*q*_ from these replicates to be close. The second measure is the standard deviation of the estimate of the slope when *C*_*q*_ is regressed against the logarithm of dilution; the smaller the standard deviation of the slope, the smaller will be that of the estimated efficiency. Following Ruitjer *et al.,* we use both of these, and compare approaches using Friedman’s non-parametric rank sum.

The three algorithms which we examine in detail, and which performed very well in the review by Ruitjer *et al.* are *Cy*0, Standard- *C*_*q*_, and PCR-Miner. The latter algorithm includes both an estimate of *C*_*q*_, and an estimate of efficiency derived from each curve, and we should emphasize that we are implementing only the *C*_*q*_-estimating component of PCR-Miner. To avoid confusion with the full PCR-Miner algorithm we will refer to it as the SDM-l5 method (second derivative maximum of the model designated l5 in the qpcR package [9] associated with the R statistical software [10]).

Notwithstanding their established utility we have concerns about each of these approaches. For *Cy*0, the derived *C*_*q*_ depends on the baseline. We regard baseline fluorescence as a ‘nuisance’ parameter, as do several algorithms that attempt to eliminate it. Our bias (and we accept that it is personal bias) is to use an estimator independent of baseline. Standard- *C*_*q*_ finds the fluorescence (*F*_*q*_) at the second derivative maximum (SDM) for the (mean) undiluted sample, and for subsequent samples *C*_*q*_ is defined as the (interpolated) cycle at which fluorescence achieves *F*_*q*_. Again this is influenced by sample-to-sample variation in baseline, and for the subsequent diluted samples it takes information from only two readings out of the entire curve. The SDM-l4 approach overcomes the above reservations by fitting a four-parameter sigmoid curve and calculating the cycle of SDM as implemented, for instance in some commercially available software [11]. This approach is independent both of baseline and of scale, but it raises a more subtle problem. Each reference curve is of a different shape. The distance between the curves in a dilution series is not well defined if each curve is of a different shape; they are not the same curve translated laterally along the dilution axis. The distance between the second derivative maxima, for instance, is different from the distance between the first derivative maxima.

## Methods

### Biological methods

Ethics approval for the use of peripheral blood leucocytes was obtained from the Human Researach Ethics Committee of The Queen Elizabeth Hospital (South Australia), and the use of samples followed the protocol approved by that committee, as documented in Bianco-Miotto *et al.* [12].

#### RNA extraction and reverse transcription

RNA was extracted from cells grown in tissue culture using Trizol (Invitrogen, USA) according to the manufacturer’s protocol. The concentration of RNA was determined using a Biophotometer (Eppendorf, North America Inc, Westbury, USA). DNAse treatment of total RNA was performed prior to reverse transcription in order to minimize PCR signal arising from carry-over genomic DNA (Ambion DNAfree kit). RNA was reverse transcribed using Superscript III RT (Invitrogen, USA). cDNA was diluted 20 fold in ultra pure water (Fischer Biotech) prior to real time PCR.

#### Preparation of genomic DNA

Mononuclear cells were isolated from the peripheral blood of healthy donors using Lymphoprep (Axis-Shield, Oslo, Norway) according to the manufacturer’s instructions. Genomic DNA (gDNA) was purified from the mononuclear cells using Trizol (Invitrogen Life Technologies, NY, USA) according to the manufacturer’s instructions.

#### Preparation of dilution series

50 *μ*l of ultra pure water was aliquoted into a series of 0.5 ml PCR tubes, and either 50 *μ*l of gDNA or 50 *μ*l of cDNA was added to the first tube and mixed by pipetting up and down 10 times. 50 *μ*l of this mixture was then pipetted to the next tube and mixed, and the process repeated across the tubes, to produce a two-fold serial dilution.

#### Real-time polymerase chain reaction

PCR amplification was performed in 20 *μ*L final volumes containing 6 *μ*L of cDNA or gDNA template, 2 *μ*L of each forward and reverse primer (5 *μ*M), and 10 *μ*L of 2 × Quantitect Sybr Green Master Mix (Qiagen, Germany). Thermocycling was performed in a Rotorgene 6000 thermocycler (Corbett, Australia) with an initial activation/denaturation (hot start) at 95°C for 15 min; followed by 45 cycles of 20 sec at 95°C, 30 sec at the annealing temperature, and 30 sec extension at 72°C. After the cycling there was a final extension at 72°C for 4 min. Triple replicates of twelve (sometimes eleven) 2-fold dilutions reactions were performed on all samples. Products were then melted in the Rotorgene 6000 thermocycler from 60°C to 99°C at 0.5°C for 5 sec per step to determine if the PCR products melted at the same temperature as PCR products that had been fractionated through 1% agarose gel to confirm that the product was of the predicted size.

Details of amplicons and primers appear under Additional file 1: Table S1.

### Numerical methods

Data analysis was carried out under GNU/Linux Ubuntu 14.04 LTS using the R programming language [10] and the associated packages qpcR [9] and ggplot2 [13]. The fixed-point estimator is as documented below. The methods Standard- *C*_*q*_, SDM-l4 nd *Cy*0 were implemented as follows.

#### Standard- *C*_*q*_

The essence of standard- *C*_*q*_ is to locate the fractional cycle corresponding to the SDM of the (averaged) undiluted sample, and to define *F*_*q*_ as the (interpolated) fluorescence at that fractional cycle. The *C*_*q*_ of each cycle, diluted or undiluted, is the fractional cycle at which *F*_*q*_ is achieved.

If *F*_*i*_ denotes the fluorescence at the *i*^*th*^ cycle of the averaged undiluted samples we find *i* for which the second derivative, (*F*_*i*−1_−2*F*_*i*_+*F*_*i*+1_) is maximal, and then assuming that the second derivative of fluorescence, if continuous, would be adequately approximated by a quadratic around the *i*^*th*^ cycle we now have as fractional cycle maximizing that quadratic as the location of SDM. The (mean) fluorescence of the undiluted sample is then found by interpolating the cubic through the adjacent four fluorescence values. This defines *F*_*q*_. For each sample we then find *k* such that *F*_*k*−1_<*F*_*q*_<*F*_*k*_ implying that *C*_*q*_ for that sample lies between (*k*−1) and *k*. Again the fractional cycle at which *F*_*q*_ occurs is found by cubic interpolation of the observed fluorescence at *F*_*k*−2_⋯*F*_*k*+1_.

#### SDM-L4

The function *pcrfit()* from the qpcR statistical package finds for each dilution curve, the parameters of best fit for the four-parameter model defined (using the nomenclature from Zhao and Ferdinand [6])

$$ y_{0} + \frac{a}{1 + (\frac{x_{0}}{x})^{b}}, $$

from which the location of the SDM is given by

$$ x_{0}\sqrt[k]{ \frac{\sqrt{3b^{2}(b^{2} - 1)} - 2(1-b^{2})}{b^{2} + 3b + 2}}. $$

#### *Cy0*

The function *pcrfit()* as above finds the parameters of best fit for a five-parameter sigmoidal curve. As introduced by Guiscini [14], the function used was

(3) $$ Fx = F_{b} + \frac{F_{max}}{(1 + e^{\beta(x_{0} - x)})^{f}},  $$

although the example in the qpcR package uses the closely related

(4) $$ Fx = F_{b} + \frac{F_{max}} { (1 + (x_{0}/x)^{\beta})^{f}},  $$

and we have implemented both for comparison. We denote the former Cy0-b5, the latter Cy0-l5, referring to the five-parameter functions b5 and l5 of the qpcR package.

The function *Cy0* from the qpcR package takes the five parameter function and returns *Cy*0 as the point of intersection with the abscissa of the tangent through the maximum first derivative.

### Theoretical development of fixed-point approach

In estimating *ΔC* we are quantifying the extent to which one curve needs to be shifted horizontally (on the cycle axis) in order that it might overlie the other. That aim requires three qualifications: first, that there may need to be some vertical shift to accommodate different baselines; second, that the same applies to scale; third that we have equally-spaced points rather than a continuous curve.

If, as in some standard analyses, the ‘position’ of a fluorescence curve is taken to be the fractional cycle at which fluorescence attains some arbitrary threshold, then the tube-to-tube variation in the baseline and scale of fluorescence becomes a problem; scale is particularly so where fluorescence has not reached a terminal plateau. The appeal of using position of maximum first or second derivative (as in PCR-Miner software) is that these are not influenced by changes in baseline or scale.

We can ask how much one fluorescence curve needs to be shifted such that it overlies another, but because we have points, rather than continuous curves we will usually find that, at best, one set of points lies close to, but between, the points of the other.

#### A reference curve

The strategy commonly adopted, and which we adopt here, is to fit our observed fluorescence data to some continuous function. The functions discussed below have in common that they are S-shaped (sigmoid). The common definition of a good fit is one which minimizes the sum of squares of differences between the observed data and the continuous function being fitted, and again we follow that practice. The qpcR package [9] uses the Marquardt-Levenberg algorithm to accomplish this. Commonly used are the five-parameter functions adopted in the variants of the *Cy*0 estimator introduced earlier simplified version of which are obtained with the constraint *f*=1, giving the four-parameter curve used in SDM-l4 or the four-parameter curve of Eq. 2 as in Liu and Saint [7]. In explaining the fixed-point approach is convenient to note that we can recast Eq. 2 as

(5) $$ Fx = Fb + \frac{F_{max}}{1 + A^{(x_{0} - x)}},  $$

where *A*=*e*^*β*^. We still have four parameters, but instead of varying *β* to obtain best fit we are varying *A*=*e*^*β*^. This makes no difference to the fit; it just makes the physical significance of the parameters more obvious to the reader.

Looking at the four parameters in turn we have

- This is the background fluorescence of an assay which we are assuming to be a nuisance variable. We want our estimate, *C*_*q*_ to be independent of *Fb*.
- This is the difference in fluorescence between *Fb* and the asymptote which fluorescence is approaching. We take tube-to-tube variation in *F*_*max*_ to result from differences in such factors as the opacity of the assay tubes; it is a nuisance variable, and *C*_*q*_ should be invariant with *F*_*max*_.
- This determines shift along the abscissa (cycle axis). In the four-parameter models mentioned, it is the fractional cycle at which the fluorescence representing reaction product is 50% of *F*_*max*_.
- This determines the shape of the curve and we note that the increase in fluorescence due to polymerase reaction during the first cycle is
  $$\frac{F_{max}}{1 + A^{(x_{0} - 1)}} \cdot \frac{1 + A^{x_{0}}}{F_{max}} = \frac{1 + A^{x_{0}}}{1 + A^{(x_{0} - 1)}}, $$
  which, for large $A^{x_{0}}\vphantom {\dot {i}\!}$ tends to *A*. That is, the parameter, *A* is the amplification efficiency during the early, exponential, part of the chain reaction.

If we now want to fit the fluorescence in each tube of a dilution series to the ‘same’ continuous function, it seems preferable to use the function which models the appropriate amplification efficiency. By the ‘same’ continuous function we mean functions for which *A* is the same. We accept that *Fb*, *F*_*max*_ and *x*_0_ will vary from tube to tube, because baseline, scale and *C*_*q*_ will vary from tube to tube. But the idea of running a dilution series to determine amplification efficiency is predicated on the assumption that amplification efficiency, and hence *A*, is the same for every tube. We might seek, therefore, to find for each tube the values for *Fb*, *F*_*max*_ and *x*_0_ that best fit the observed fluorescence in that tube, keeping *A* fixed at the amplification efficiency as derived in the usual way from a regression of *C*_*q*_ against logarithm of dilution.

The impasse is now obvious; the point of dilution assay is to determine the amplification efficiency. Until we know the amplification efficiency we do not know the appropriate value of *A* to use in Eq. 5 to determine the *C*_*q*_ for each tube. The Bauer fixed-point theorem resolves that impasse. Using fixed-point convergence (see, for instance, [15]), we begin with an initial guess, *A*_0_. This leads to estimates *C*_*q*_ for each tube on the basis of which the slope of regression against logarithm of dilution gives a first estimate *E*_1_ of amplification efficiency. We now replace our initial guesstimate *A*_0_ with *A*_1_=*E*_1_ and repeat the process giving a second estimate *E*_2_ and so on, until subsequent estimates are unchanged and convergence has been achieved.

For the process to converge, the requirement of the fixed-point theorem is that a plot of *E*_1_ against *A*_0_ (which is, of course also that of *E*_2_ against *A*_1_ and so on), should have an absolute slope less than one. Providing this condition is satisfied (and for the data sets considered here it is), the theorem asserts that

- the process will converge
- the smaller the slope, the faster it will converge
- the value to which it converges is independent of the starting estimate *A*_0_

We note that, in fitting Eq. 5 to an observed fluorescence curve, if *A* is fixed, there are only three parameters, *Fb*, *F*_*max*_ and *x*_0_ of which both *Fb* and *F*_*max*_ enter the equation linearly. Since we are really only interested in the parameter *x*_0_ we can, under these circumstances, avoid using the Marquardt-Levenberg algorithm and find the value of *x*_0_ which maximizes the Pearson correlation coefficient between observed fluorescence and the fitted curve. That correlation will be independent of *Fb* and *F*_*max*_, and will give the same ‘best fit’ *x*_0_ as a Marquardt-Levenberg approach minimizing the sum of squares of differences. If we denote by *x*_*i*_ the value of *x*_0_ maximizing correlation in the *i*^*th*^ tube, each tube now has a reference curve of the form

$$ \frac{1}{1 + A^{(x_{i} - x)}}. $$

The reference curves for all tubes are now the same shape, apart from their shift along the abscissa determined by *x*_*i*_. Because shift along the abscissa is the only difference between any two curves the concept of *ΔC*_*q*_ as that difference is now unambiguous; we could define it as the difference between first derivative maximum, second derivative maximum, or difference between cycles at which some fraction of the increase has been achieved and all these definitions would result in the same *ΔC*_*q*_. The simplest definition is to use *x*_*i*_ as the *C*_*q*_ for the corresponding tube (the cycle at which the reaction is 50% of its maximum fluorescence).

In summary the steps in the fixed-point algorithm are as follows. We will use the publicly available dataset *batsch1* from the qpcR package as an illustration, including the numerical values which we obtain.

**Figure 1:**
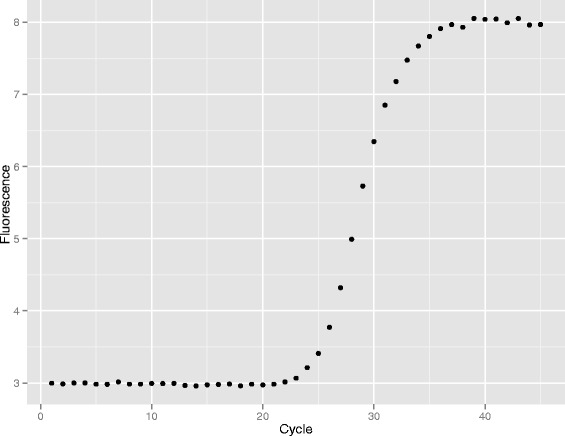
**A Fluorescence Curve.** The first fluorescence curve from the *batsch1* data set is approximately half-way between baseline and its maximum at cycle 29. We expect, therefore, that correlation with the sigmoid reference curve will maximize at a value of *x*_0_≈29.

1. Using an initial guesstimate of amplification efficiency (we have used *A*=1.5) define
  $$ f(A, x_{0}, x) = f(1.5, x_{0},x) = \frac{1}{1 + 1.5^{(x_{0} - x)}}. $$
2. Use algorithm of choice to find, for each tube, the value of *x*_0_ which maximizes the correlation between *f*(*A*,*x*_0_,*x*) and the fluorescence data from that tube. For that tube let *C*_*q*_=*x*_0_. The first fluorescence data from *batsch1* is shown in Figure 1. By inspection we can see that half the generated fluorescence occurs by about cycle 29, so the reference curve showing greatest correlation will have a value of *x*_0_ of about 29. Figure 2 shows a plot of correlation against *x*_0_ for values of *x*_0_ from 1 to 45 and we can see the correlation maximizing at cycle 29. A good first-estimate of the fractional cycle at which correlation maximizes is obtained by quadratic interpolation using the three correlations at this and the adjacent cycles. Given the correlations are 0.99497, 0.99766, and 0.99408 at cycles 28, 29 and 30 respectively we imply a maximum at cycle
  $$ x_{0} = \frac{0.99497 - 0.99408}{2(0.99497 - 2 \times 0.99766 + 0.99408)} = 28.929. $$

**Figure 2:**
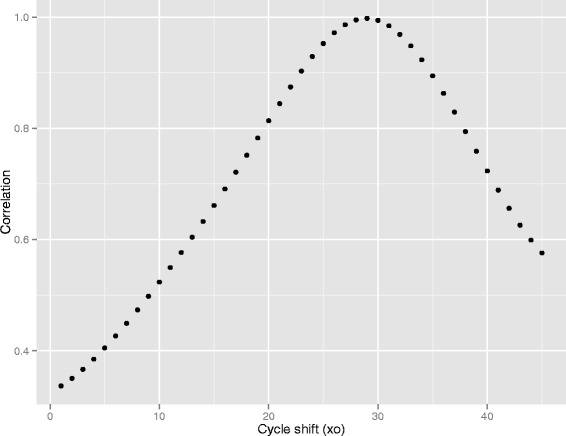
**Correlation between reference curve and data.** The correlation between the reference curve and the data of Figure 1 maximizes at a value *x*_0_≈29. The value of the fractional cycle maximizing correlation can be found either by fitting a quadratic through 29 and the adjacent points, or by a one-dimensional non-linear search. For the illustrated data, the maximum is at 28.93 cycles.
  Of course, any iterative approach can be used, and as we are only looking for a maximum in one dimension the R procedure *optimize()* is appropriate. In practice, for these data there is no improvement; a maximum correlation is found at cycle fractional cycle 28.930. Set *C*_*q*_ for this tube as 28.93. Repeat for each tube.
3. Regressing *C*_*q*_ against logarithm of dilution determine estimated amplification efficiency, and return to step 1 replacing *A* with this estimate. We prefer to use logarithms to base 2 as in Figure 3 because the implication of the regression slope is clear from inspection; a doubling at each cycle (*E*=2) would imply that a two-fold dilution shifts the fluorescence curve by exactly one cycle. The regression slope in Figure 3 is 1.185, implying that it takes 1.185 cycles to compensate for a two-fold dilution. If there is an *E*-fold increase each cycle then
  $$E^{1.185} = 2 $$
  from which it is immediate that
  $$E_{1} = \sqrt[1.185]{2} = 1.795. $$

**Figure 3:**
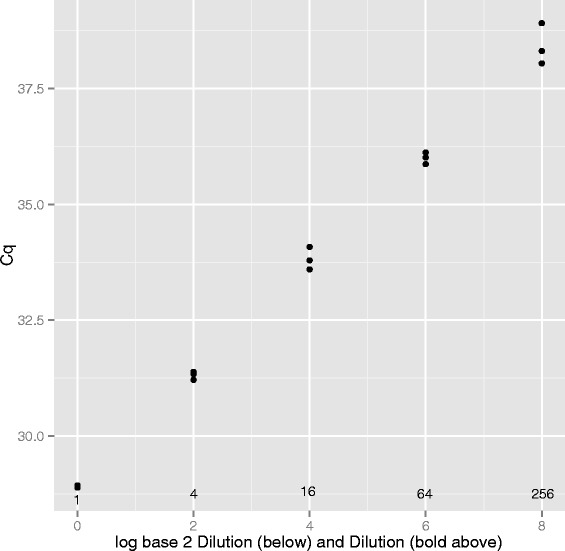
**The regression of** ***C***_***q***_ **against logarithm of dilution (base2).** If each PCR cycle were to double the DNA, then a two-fold dilution would delay the curve by one cycle. The slope of the straight line regressing *C*_*q*_ against log of dilution (base 2) tells us how many cycles are required to double the DNA. Using a reference curve with *A*=1.5 slope is 1.185 cycles per doubling, or equivalently increasing the DNA 1.795 - fold per cycle. The next fixed-point iteration will use *A*=1.795 in the reference curve.
4. Return to step 1, replacing the initial *A*=1.5 with *A*=1.795. Iterate until estimated efficiency is unchanged. This is the fixed-point iteration, and is illustrated in Figure 4. In the above we started with guesstimate *A*_0_=1.5 (deliberately far from what we expect, so as to illustrate the method) and the first cycle returns an estimated efficiency 1.795. This corresponds to the vertical blue line on Figure 4 in which the red curve shows for this process the output *E* for a range on input *A* from 1·4 to 2·2. Bauer’s fixed point theorem guarantees convergence if the slope of this line is absolutely less than one in the region of interest (as shown here for these data). We then replace our initial *A*_0_=1.5 with the revised *A*_1_=1.795 (horizontal blue line, completing the first iteration of fixed-point convergence. The second cycle is shown in green, giving the second estimate *E*_2_=1.848 and so on, converging rapidly to an amplification efficiency of 1.8517.

**Figure 4:**
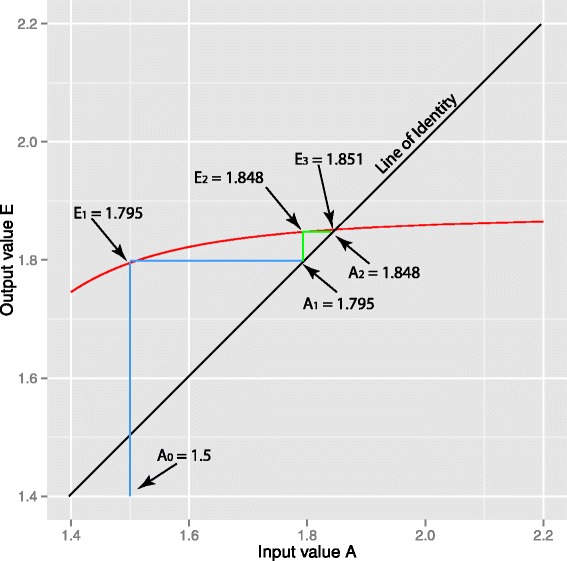
**Fixed-point iteration applied to the batsch1 data set.** The estimated amplification efficiency, (E - out, vertical axis), varies according to the assumed amplification efficiency (A - In, horizontal axis) used to construct the sigmoid reference curve. The red line is a plot of (E - out) as a function of (A - In). Applied iteratively, however, the process converges to the point of intersection of the red curve and the line of identity (black). This point of convergence, the fixed-point, is independent of the starting value. With a starting value of *A*=1.5 the first iteration is shown in blue, the second in green.

## Results and discussion

### Fixed point convergence

**Figure 5:**
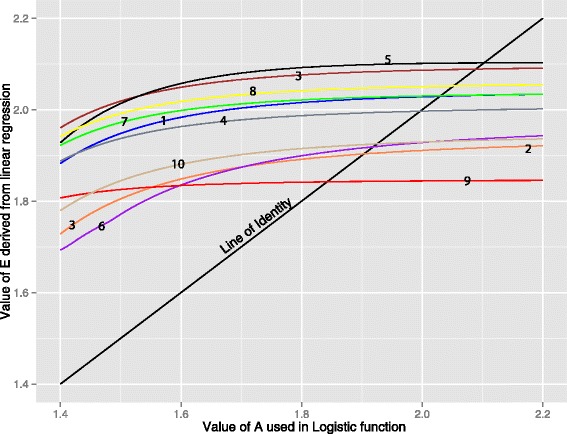
**Convergence of Fixed-point Iteration.** The convergence of the iterative process illustrated in Figure 4 depends on the slope of the curve relating E - out to A - In. The process will converge provided the slope is not too great (Bauer’s fixed-point theorem). We illustrate the ten data sets from this laboratory: the slope is less than that of the line of identity, and fixed-point convergence is guaranteed. Numbers 1 to 10 correspond to the data sets Flind1 - Flind10 respectively.

For all data sets with an initial estimate *A*_0_=1.5 used in the logistic function, deliberately far from the expected *E*, the fixed-point iteration had converged to within.001 of its asymptote in four iterations; a convergence well beyond any biological relevance. The rapid convergence results from the relative insensitivity of estimated *E* to the *A* used in the logistic reference curve. Figure 5 shows estimated *E*_1_ as a function of input *A*_0_ for all ten data sets from our laboratory. The slope of all curves in the interval of interest is well within the (−1,1) required for convergence by the Banach fixed-point theorem. For nine of the ten data sets, however, the parameter of the logistic function does influence the corresponding *E* estimate and it is important that the parameter be determined objectively. Additional files 2 and 3 show the corresponding figures for the publicly available data sets.

There are 23 data sets, giving 23 standard deviations for estimates of slope by each of the five analysis methods. The rank sums appear in the first line of Table 1. Using Friedman’s rank sum to compare the standard deviation of the estimate of slope the null hypothesis of equal distributions is rejected (*χ*_4_^2^=25.7,*p*<0.0001). The best two estimators are SDM-l4 and Fixed-point. Proceeding to a direct comparison of the two, the Fixed-point estimator performs better, having a smaller standard deviation in 15 of 23 comparison. The difference, however, is not of statistical significance.

**Table 1:** **Sum of ranks (smaller is better)**

**Variable**	**SDM-l4**	**Std** ***C***_***q***_	**Cy0-l5**	**Cy0-b5**	**Fixed point**
Replicates sd	388	669	574	662	422
Slope sd	46	96	68	83	52

For each of the five estimators of ***C***_***q***_ we have ranked their performance as judged by the standard deviation of replicates (181 sets of replicates, line 1) and by the standard deviation of the estimated slope when ***C***_*q*_ is regressed against log. dilution (23 data sets, line 2). For both approaches the null hypothesis of equal distributions of ranks is rejected. On direct comparison of the best two (Fixed-point *vs* SDM-l4), Fixed-point has a smaller standard deviation of replicates in 95 of 181 comparisons, and a smaller standard deviation of slopes in 14 of 23 comparisons.

Within the 23 data sets are 181 sets of replicate *C*_*q*_. The rank sums of replicate standard deviations appear as the second line of Table 1. Again using Friedman’s rank sum test on the null hypothesis of equal distributions of replicate standard deviations the null hypothesis is rejected (*χ*_4_^2^=149,*p*<0.0001). On direct comparison of the better two estimators, SDM-l4 and Fixed-point, the Fixed-point is the better estimator in 96 of 181 comparisons but again this is not statistically significant.

### Caveats

**Figure 6:**
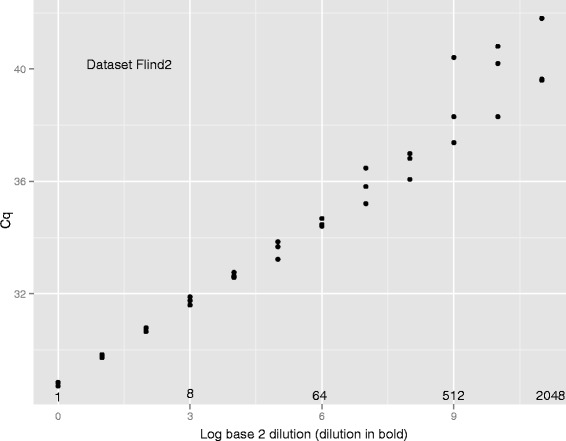
**Regression of** ***C***_***q***_ **against logarithm of dilution.** Amplification efficiency is estimated from the slope of the line relating *C*_*q*_ to the dilution, the latter on a logarithmic scale. The confidence limits for the slope, when calculated from unweighted least-squares (as commonly done in simple linear regression) is strictly appropriate only for normally-distributed homoscedastic data. We caution that our data are not homoscedastic, and are unlikely to be normally distributed.

We would caution, however, against too literal an interpretation of the standard deviation of the slope estimate. The linear regression of position against log(*dilution*) on which it is based assumes normally-distributed homoscedastic data. Data from qPCR dilution series are almost never homoscedastic because higher dilutions lead to more variable fluorescence sequences than do lower dilutions. Even if errors at low dilutions are Gaussian, those at higher dilutions result from Poisson effects and will not be Gaussian. Figure 6 is characteristic of the regression line and associated data, and demonstrates the increased variance at higher dilutions. In addition, the linear regression assumes that the dilution itself is error-free, and we doubt that this is true of our own data. Although these concerns lead us to doubt the absolute values of the standard deviations, the concerns apply equally to the five estimators, and are unlikely to draw into question the ordering of these for any one data set.

When *C*_*q*_ is regressed against log dilution the standard deviation of the slope depends in part on whether *C*_*q*_ increases linearly with log dilution, and this in turn depends on amplification efficiency being constant at all dilutions. In using the variance of slope as a measure of the merit of a method, we assume that amplification efficiency is invariant with dilution. In practice the data sets we have analyzed show a remarkable linearity; the reason for assaying at intermediate dilutions is to confirm that linearity, without which a dilution series would be difficult to interpret.

The fixed-point method assumes that the fluorescence data approach the plateau. If, in a dilution series, the higher dilutions result in only the very early part of the fluorescence curve emerging, then the estimated *C*_*q*_ at these dilutions will be unreliable.

Finally we have presented a comparison of the five methods discussed, and use Friedman’s non-parametric rank sum as a test of the null hypothesis that the methods are equivalent. Our data, however, are not randomly selected from the population of dilution series in general, and the Friedman’s test should be interpreted with caution in this context. We have examined two ‘merits’ of the methods: replicate standard deviation and slope standard deviation. These are not independent: the standard deviation of the slope estimate takes into account that of the replicates.

## Conclusion

The use of a reference curve, (in this case logistic) relative to which the position of fluorescence data can be measured, avoids subjective decisions as to baseline and scale and threshold. Using data from the whole curve, rather than just a few points, it offers an approach to the estimation of amplification efficiency from a dilution series. The logistic function represents a family of curves, however, and the specific curve appropriate to a given dilution series can be defined by fixed-point iteration. Convergence is rapid and for the illustrative data used here the method is often, but not always, an improvement on existing estimators.

## Additional files

Additional file 1
**Table S1.** Details of Incubates Flind1 to Flind10.

Additional file 2
**Convergence of Fixed-point Iteration.** This figure corresponds to Figure 5 of the main text, but looks at the publicly-available data sets batsch1 to batsch5 (blue, brown, red, purple, black) and reps (turquoise), reps2 (green), reps3 (yellow).

Additional file 3
**Convergence of Fixed-point Iteration.** As for Additional file 2, but with the publicly-available data sets boggy (blue), guiscini (brown), lievens1 (red), rutledge (purple) and sisti (green).
