# Supplementary material for: A fixed-point algorithm for estimating amplification efficiency from a polymerase chain reaction dilution series
Source: BMC Bioinformatics. 2014 Dec 10;15(1):372. doi: 10.1186/s12859-014-0372-4 (PMC4268849; doi:10.1186/s12859-014-0372-4)
Supplement: Additional file 1 — Table S1. Details of Incubates Flind1 to Flind10. [file 12859_2014_372_MOESM1_ESM.pdf]

Table S1 Details of Incubates Flind1 to Flind10. PBL - peripheral blood lymphocytes. HT29 - a cell line derived from colon malignancy.

File	DNA	Tissue	Amplicon	Primers (forward above reverse)
Flind1	genomic	PBL	GADD153	CTGCACCAAGCATGAACAAT<br>GGGATTGAGGGTCACATCAT
Flind2	genomic	PBL	Cox-1	CACATGACTACATCAGCTGGGAGT<br>GTGGGGCAATCTTTAGGCACAGAG
Flind3	genomic	PBL	h beta-actin	TTGCCGACAGGATGCAGAAG<br>GCCGATCCACACGGAGTACT
Flind4	genomic	PBL	h beta-actin	TTGCCGACAGGATGCAGAAG<br>GCCGATCCACACGGAGTACT
Flind5	genomic	PBL	UGT-2B4	TTACGTCTGAGGCTGGAAGC<br>TGTTTTGTCACAAGAAGAAAGGA
Flind6	genomic	PBL	p52	CTGACTCAGACTGACATTCTCCAC<br>TTCTGACGCACACCTATTGCAAGC
Flind7	comp	HT29	c-myc	CCTCACAACCTTGGCTGAGT<br>GTTCTTTTATGCCCAAAGTCCA
Flind8	comp	HT29	c-myc	CCTCACAACCTTGGCTGAGT<br>GTTCTTTTATGCCCAAAGTCCA
Flind9	comp	HT29	GADPH	TGCACCACCAACTGCTTAGC<br>GGCATGGACTGTGGTCATGAG
Flind10	genomic	PBL	Cox-1	CACATGACTACATCAGCTGGGAGT<br>GTGGGGCAATCTTTAGGCACAGAG
