# Supplementary material for: A fixed-point algorithm for estimating amplification efficiency from a polymerase chain reaction dilution series
Source: BMC Bioinformatics. 2014 Dec 10;15(1):372. doi: 10.1186/s12859-014-0372-4 (PMC4268849; doi:10.1186/s12859-014-0372-4)
Supplement: Additional file 2 — Convergence of Fixed-point Iteration. This figure corresponds to Figure 5 of the main text, but looks at the publicly-available data sets batsch1 to batsch5 (blue, brown, red, purple, black) and reps (turquoise), reps2 (green), reps3 (yellow). [file 12859_2014_372_MOESM2_ESM.pdf]

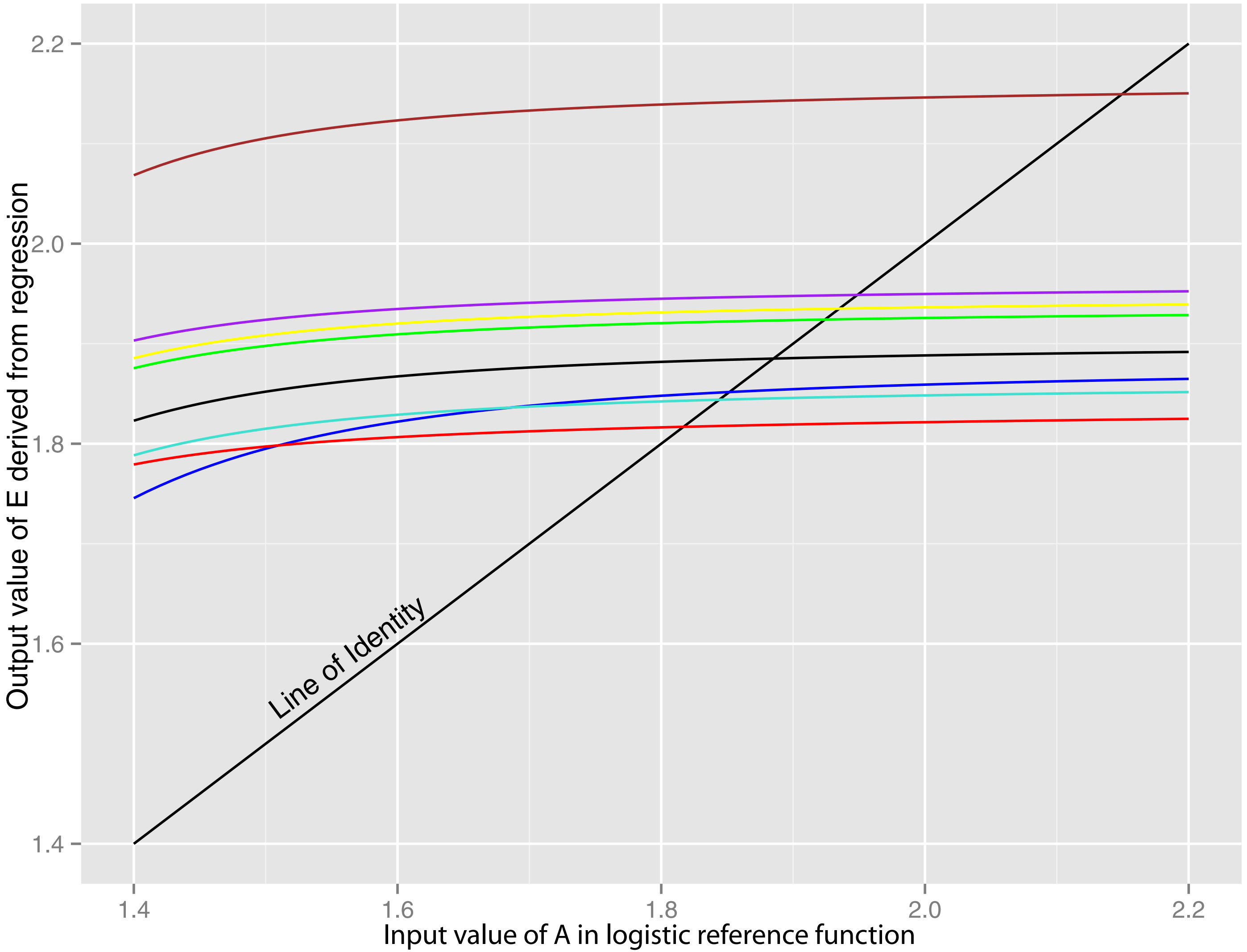

Output value of E derived from regression
Input value of A in logistic reference function
Line of Identity
2.2
2.0
1.8
1.6
1.4
1.4
1.6
1.8
2.0
2.2
