# Supplementary material for: A fixed-point algorithm for estimating amplification efficiency from a polymerase chain reaction dilution series
Source: BMC Bioinformatics. 2014 Dec 10;15(1):372. doi: 10.1186/s12859-014-0372-4 (PMC4268849; doi:10.1186/s12859-014-0372-4)
Supplement: Additional file 3 — Convergence of Fixed-point Iteration. As for Additional file 2, but with the publicly-available data sets boggy (blue), guiscini (brown), lievens1 (red), rutledge (purple) and sisti (green). [file 12859_2014_372_MOESM3_ESM.pdf]

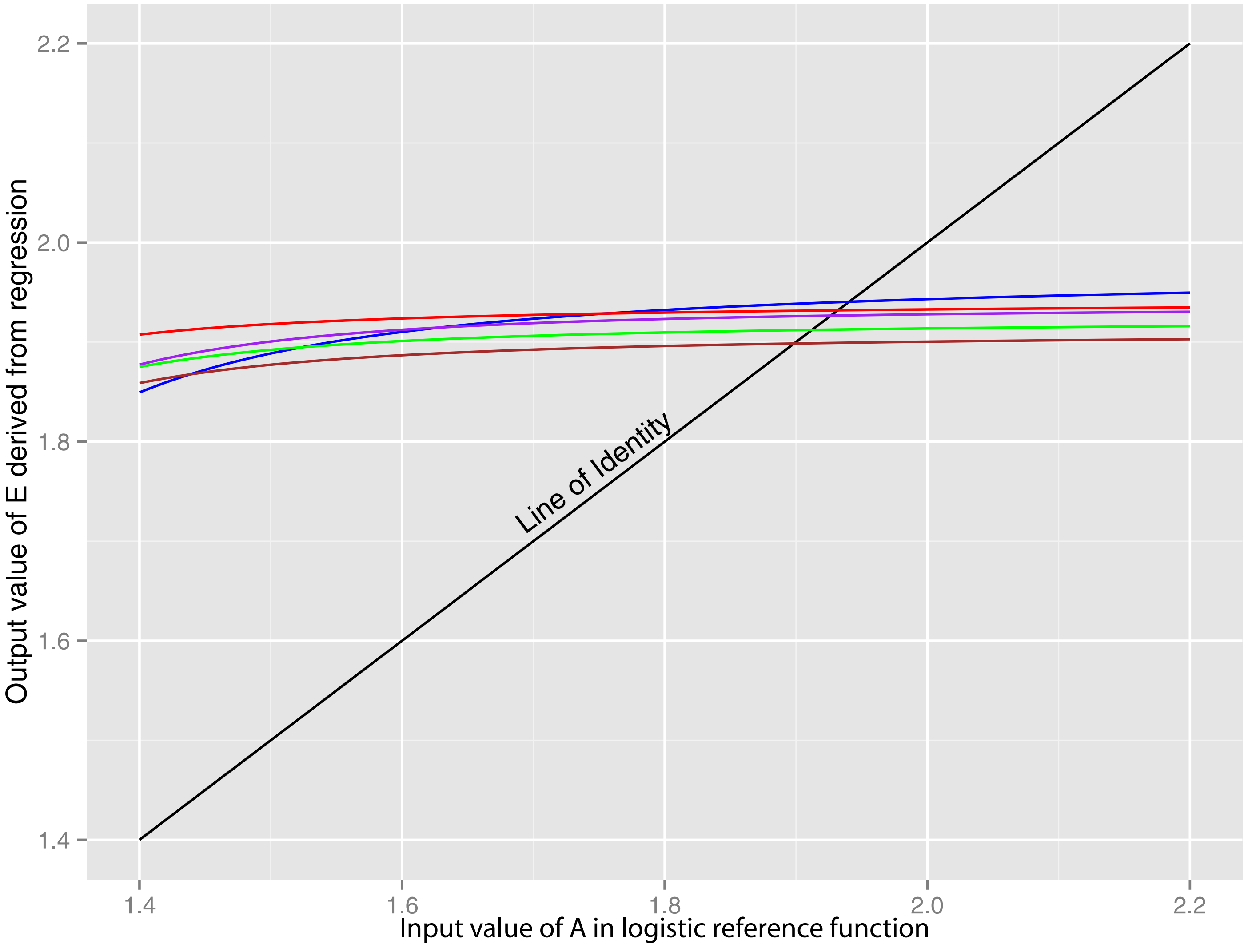

Output value of E derived from regression
2.2
2.0
1.8
1.6
1.4
Line of Identity
1.4
1.6
1.8
2.0
2.2
Input value of A in logistic reference function
